# Progression from prediabetes to type 2 diabetes mellitus in adolescents: a real world experience

**DOI:** 10.3389/fcdhc.2023.1181729

**Published:** 2023-05-09

**Authors:** Alyson Weiner, Meng Zhang, Sheng Ren, Beverly Tchang, Rachelle Gandica, Jaime Murillo

**Affiliations:** ^1^ Comprehensive Weight Control Center, Division of Endocrinology, Diabetes, and Metabolism Weill Cornell Medicine, New York, NY, United States; ^2^ Optum Labs, Eden Prairie, MN, United States; ^3^ Division of Pediatric Endocrinology, Diabetes, and Metabolism Columbia University Irving Medical Center, New York, NY, United States

**Keywords:** prediabetes, adolescents, metformin, type 2 diabetes, pediatric diabetes care

## Abstract

**Background:**

Obesity in pediatric patients is strongly associated with increased vascular and metabolic risk. Prediabetes is present in up to 1 in 5 adolescents, aged 12-18 years-old, though is thought to remit spontaneously in a significant portion. Pediatric patients with type 2 diabetes mellitus (T2D) have a more rapid decline of beta-cell function and progression to treatment failure than adult T2D patients. Thus, there is a strong interest in better understanding the natural history of prediabetes in these youth. We aimed to evaluate the real-world rate of progression of prediabetes to T2D in adolescent patients.

**Methods:**

This is a retrospective study of 9,275 adolescent subjects aged 12-21 years-old with at least 3 years of de-identified commercial claims data and a new diagnosis of prediabetes during the observation period. Enrollees with a T2D diagnosis and/or diabetes medication use in the 1 year prior to prediabetes diagnosis or a T2D diagnosis in the 1 month following prediabetes diagnosis were excluded. Enrollees with diagnoses of type 1 diabetes (T1D) or polycystic ovarian syndrome over the 3 years were also excluded. Progression to T2D was defined by claims data of two T2D diagnoses at least 7 days apart, HbA1c ≥ 6.5%, and/or prescription of insulin without known T1D. Enrollees were followed for 2 years after prediabetes diagnosis.

**Results:**

Overall, 232 subjects (2.5%) progressed from prediabetes to T2D. There were no differences found in T2D progression based on sex or age. Progression to T2D occurred at a median of 302 days after prediabetes diagnosis (IQR 123 to 518 days). This study was limited by the lack of laboratory/anthropometric data in administrative claims, as well as the exclusion of 23,825 enrollees for lack of continuous commercial claims data over 3 years.

**Conclusion:**

In the largest sample to date on adolescent prediabetes, we found a 2.5% progression of prediabetes to T2D over a median duration of about one year.

## Introduction

In the United States, the prevalence of obesity in children and adolescents was 17% between 2011-2014, with 5.8% having extreme obesity (body mass index (BMI) ≥ to 120% of the 95th%ile for age and sex or ≥35kg/m^2^) (1). Obesity in pediatric patients is associated with increased vascular and metabolic risk (hypertension, coronary artery disease, type 2 diabetes mellitus (T2D), dyslipidemia, and hepato-steatosis) (2).

Prediabetes is defined as impaired fasting glucose, impaired glucose tolerance, or elevated glycated hemoglobin (HbA1c) between 5.7% and 6.4%, and is present in up to 1 in 5 adolescents, aged 12-18 years-old (3). T2D results from beta-cell dysfunction and a decline in peripheral and hepatic insulin sensitivity, and is associated with long-term microvascular and macrovascular complications (4). In the Treatment Options for T2D in Adolescents and Youth (TODAY) cohort study, adolescents with T2D were found to have cardiometabolic comorbidities soon after diagnosis (5). Furthermore, by the time of diagnosis with T2D, beta-cell function and insulin secretion were significantly impaired, with residual beta-cell function as a major predictor of glycemic control in pediatric patients with recently diagnosed T2D (6, 7). The prevalence of T2D in pediatric patients in the United States varies by race and ethnicity, though is highest in Native Americans (8).

Adolescents with prediabetes may revert to normoglycemia on repeat testing, which is thought to be related to an improvement in insulin resistance after puberty (9). The progression of prediabetes to T2D is not well-characterized in adolescents, with a wide range reported in published studies of those who progress: 2-24% (10–12). As such, we aim to evaluate the real-world progression of prediabetes to T2D in a large cohort of adolescents with de-identified commercial claims data.

## Materials and methods

### Study population

This study used de-identified administrative claims data from the Optum Labs Data Warehouse (OLDW), which includes medical and pharmacy claims, laboratory results, and enrollment records for commercial and Medicare Advantage (MA) enrollees. The database contains longitudinal health information for over 200 million enrollees and patients, representing a mixture of ages and geographical regions across the United States.

Enrollees aged 12-21 years with a new diagnosis of prediabetes between 2010 and 2017 were initially screened for this retrospective analysis. Data were de-identified. Prediabetes diagnosis was defined by one claim for an ICD9 or ICD10 claim consistent with the diagnosis (**Table 1**). The index date was defined as the first claim for prediabetes. Enrollees were required to have at least three years of continuous claims data (one year prior to index date and two years post-index date) for both medical and pharmacy claims. Enrollees missing age, sex, and/or region were excluded.

**Table 1:** Diagnosis codes.

ICD-9 DX	790.21	Impaired fasting glucose
ICD-9 DX	790.22	Impaired glucose tolerance
ICD-9 DX	790.29	Other abnormal glucose
ICD-10 DX	R73.03 (effective 2017)	Prediabetes
ICD-10 DX	R73.01	Impaired fasting glucose
ICD-10 DX	R73.02	Impaired glucose tolerance
ICD-10 DX	R73.0	Abnormal glucose
ICD-10 DX	R73.09	Other abnormal glucose
ICD-10 DX	R73.9	Hyperglycemia, unspecified

Enrollees with a T2D diagnosis and/or diabetes medication use (metformin, insulin, glucagon-like peptide-1 receptor agonists (GLP-1 RAs), sodium/glucose cotransporter-2 inhibitors (SGLT2i), sulfonylureas, thiazolidinediones, dipeptidyl peptidase 4 inhibitors (DPP4i), and metformin dual therapy) in the one year prior to prediabetes diagnosis were excluded. To limit erroneous prediabetes claims, enrollees with a T2D diagnosis or a prescription of a second-line diabetes medication in the 30 days following prediabetes diagnosis were not included for analysis. Enrollees with diagnoses of type 1 diabetes mellitus (T1D) or polycystic ovarian syndrome (diagnosis codes for polycystic ovarian syndrome, irregular periods, androgen excess, and oligomenorrhea) over the three years were also excluded.

Subjects were followed for a total of 24 months after prediabetes diagnosis.

Because data were de-identified in compliance with the Health Insurance Portability and Accountability Act and customer requirements, Institutional Review Board approval or waiver of authorization was not required.

### Clinical assessments/outcomes

Progression to T2D was defined by claims data of two T2D diagnoses at least seven days apart, HbA1c **≥** 6.5%, and/or prescription of insulin without known T1D.

Claims for the prescription of metformin and second-line diabetes medications between the index date and 2 years post-index date were evaluated.

At least one HbA1c laboratory value was available in 2,983 subjects in this study. However, the majority of these subjects only had 1 value reported. Given the limited HbA1c data, this value was not used for the diagnosis of prediabetes, but was used, when available, for the diagnosis of T2D at follow-up.

## Results

### Baseline characteristics

**Figure 1:**
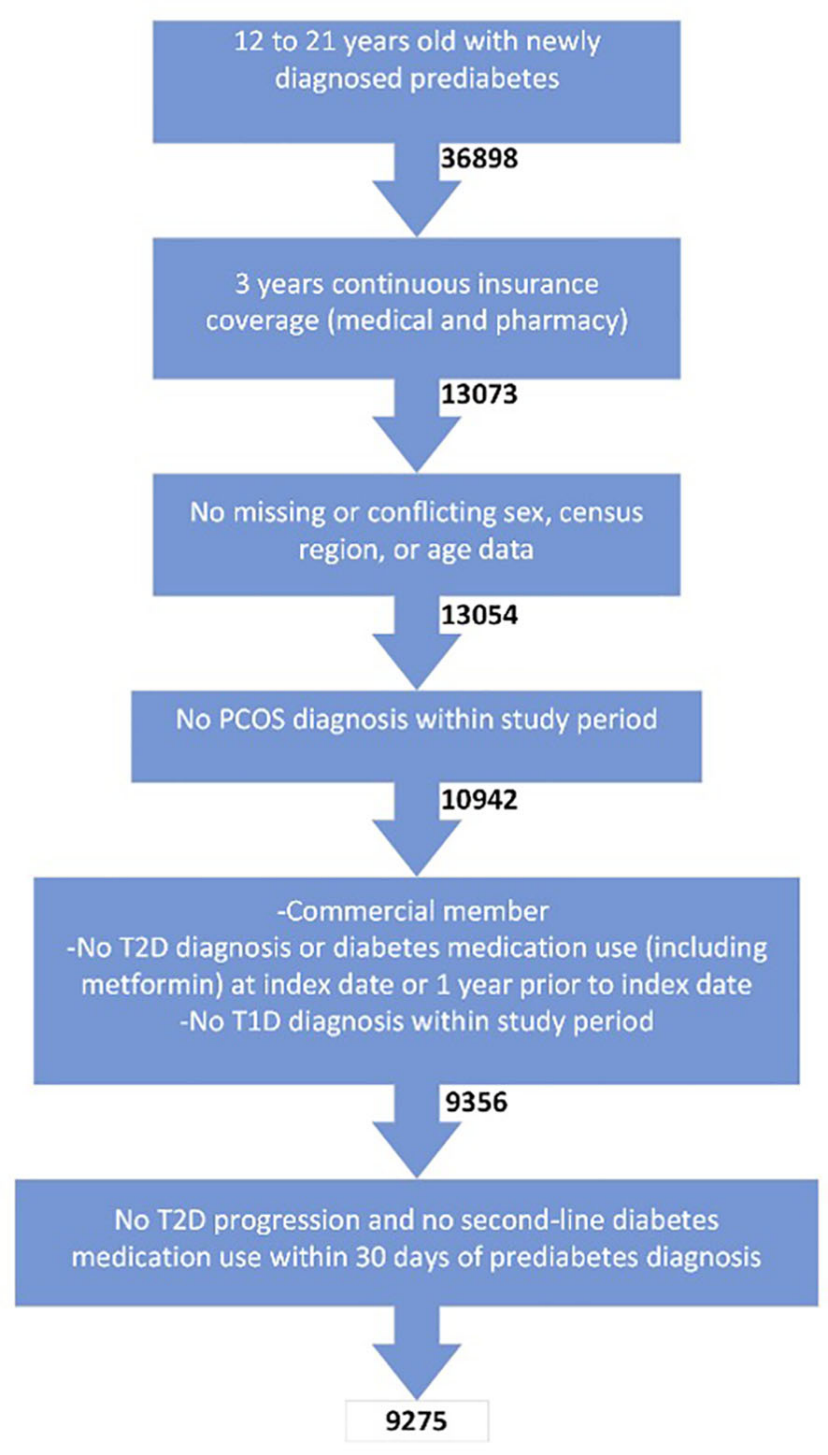
Inclusion criteria for final cohort.

Of the 36,898 enrollees aged 12-21 years old with prediabetes screened for inclusion criteria, 9,275 subjects were included in the final cohort (**Figure 1**). The main reason for exclusion was the lack of claims data over the 3-year period.

### Progression to T2D

Of 9,275 subjects with incident prediabetes at the start of study, 232 (2.5%) progressed from prediabetes to T2D over the 2-year follow-up period. There were not any differences in T2D progression based on sex or age (**Table 2**). Progression to T2D occurred at a median of 302 [IQR 123, 518] days after prediabetes diagnosis.

**Table 2:** Baseline Demographic Information.

		N= 9275
**Age (years)^1^**		18 (15,20)
**Age Distribution**	Age 12-17	4625 (49.87%)
	Age 18-21	4650 (50.13%)
**Sex**	Female	4570 (49.27%)
	Male	4705 (50.73%)
**Region**	Northeast	920 (9.92%)
	Midwest	2095 (22.59%)
	South	4683 (50.49%)
	West	1577 (17.0%)

1. Data listed as median (IQR).

### Use of medications

Only 4.32% of the subjects with prediabetes were placed on metformin, with 0.71% prescribed a second-line diabetes medication (**Table 3**). The most common second-line medication prescribed was insulin, followed by sulfonylureas and then GLP-1 RAs. As expected, second-line diabetes medications were more frequently prescribed in the 18-21 year-old cohort.

**Table 3:** T2D diagnosis and diabetes medication prescriptions within 24 months of prediabetes diagnosis.

	N=9275	Age 12-17 N=4625	Age 18-21 N=4650
**T2D Diagnosis (excluding T2D in first 30 days after prediabetes diagnosis)**	232 (2.5%)	110 (2.38%)	122 (2.62%)
**Metformin Prescribed**	401 (4.32%)	243 (5.25%)	158 (3.40%)
**At Least 1 Second-line Diabetes Medication Prescribed**	66 (0.71%)	25 (0.54%)	41 (0.88%)
**Insulin Prescribed**	28 (0.3%)	14 (0.30%)	14 (0.30%)
**GLP-1 RA, DPP4i, or SGLTi Prescribed**	26 (0.28%)	6 (0.13%)	20 (0.43%)
**Sulfonylurea, Thiazolidinedione, or Metformin Combination Therapy Prescribed**	26 (0.28%)	5 (0.11%)	21 (0.45%)

## Discussion

Our study of over 9,275 adolescents is the largest published cohort to examine the progression from prediabetes to T2D, finding a 2.5% progression over a median duration of about one year. One study of 488 pediatric subjects with prediabetes found that 8% of subjects with a baseline HbA1c of 6-6.4% and 4% of those with a baseline HbA1c of 5.7-5.9% had a HbA1c in the T2D range at a median follow-up of 12-22 months (12). Unlike our study, this was the experience of a single urban health-care system (Denver Health and Hospitals), and subjects with PCOS do not appear to have been excluded. In another study of 128 children with obesity and impaired glucose tolerance (IGT), 96 (75%) reverted to normal glucose metabolism, 20 (16%) still had IGT, while 3 (2%) developed T2D in the subsequent 3-5 years (10). A third study of 162 youth with IGT at baseline found that 105 (65%) reverted to normal glucose tolerance, 44 (27%) had persistent IGT, and 13 (8%) progressed to T2D at a median of 2.9 years of follow-up (13). Conversely, one small study of 33 youth (mean age 12.5 years at baseline) with IGT found that 8 (24.2%) progressed to T2D, while 15 (45%) had normal glucose tolerance after 2 years (11). Overall, existing studies of prediabetes-to-T2D progression in the pediatric and adolescent populations report a progression of 2-24%, a range that is likely attributable to small sample sizes and variations in study design (10–12).

Predictors of progression have been investigated in prior studies. BMI stabilization was associated with decreased risk of T2D progression (12), while a higher BMI and Black race were associated with progression to T2D (11). There is evidence from other pediatric studies that various factors, including family history of T2D, maternal gestational diabetes, continued weight-gain, and race/ethnicity, influence the progression of prediabetes to T2D.

The degree of progression from prediabetes to T2D appears to be lower in the pediatric population, compared to adults. It is estimated that about 5-10% of adults with prediabetes develop diabetes each year, though the ADA estimates up to 70% of adults with prediabetes will develop T2D (14). Although the rate of progression from prediabetes to T2D in adolescents is not well-characterized, it may occur faster in pediatric patients, as compared to adult patients (15).

In this real-world analysis, 4.32% of the subjects were placed on metformin, though it is not clear from the available data if this medication was prescribed off-label for prediabetes or if the medication was prescribed after progression to T2D. Information on adherence was not assessed during this study. As such, we were unable to evaluate if the use of metformin influenced the progression of prediabetes to T2D. Metformin is recommended for prediabetes in adults, though not in pediatrics (16). Metformin is generally well-tolerated and primarily acts in the liver to reduce hepatic glucose production and peripherally to improve insulin sensitivity. Metformin also acts to increase circulating levels of glucagon-like peptide-1 to enhance glucose-dependent insulin release from the pancreas (17). It is FDA approved for children ≥10 years of age as first line medical therapy (in addition to lifestyle interventions) for metabolically stable pediatric patients with T2D (18). However, off-label use of metformin is prevalent for pediatric metabolic syndrome, PCOS, and obesity (19). Studies on metformin in pediatric subjects without T2D have shown inconsistent results of metformin on BMI, cardiometabolic outcomes, and development of T2D, though have utilized varying metformin doses and lengths of follow-up (20–24).

We also evaluated second-line diabetes medication prescriptions, though the specific diagnoses claims associated with the prescriptions are also unknown. Insulin was most commonly prescribed, followed by sulfonylureas and then GLP-1 RAs. This is similar to the findings of a recent study of 829 adolescents (10-18 years-old) with T2D prescribed metformin. 207 subjects (25%) required treatment escalation within 5 years of diagnosis, and insulin was most commonly prescribed for escalation, followed by sulfonylureas, and then by GLP-1 RA (25). In patients <18 years-old, metformin and insulin were the only approved therapies for T2D, until liraglutide (daily GLP1-RA) was approved for use in patients (≥ 10 years old) in 2019. In our study, the prescription of any second-line medications, besides insulin, in the 12-17 year-old cohort would be considered off-label. Based on ADA guidelines for T2D in adults, second-line medication therapy (after metformin) should be individualized. GLP-1 RA and SGLT2i may be considered if a patient is high risk or has established atherosclerotic cardiovascular disease, chronic kidney disease, or heart failure. If HbA1c is above target, DPP4-i, GLP-1 RA, SGLT2i, thiazolidinediones, and sulfonylureas may be considered (26). The limited use of these glycemic agents, as compared to insulin, may have been due to challenges with insurance authorization or provider preference. Although enrollees with a diagnosis code of T1D were excluded, we cannot rule out the possibility that some of the subjects on insulin were incorrectly diagnosed or coded as T2D and then appropriately placed on insulin therapy. Of note, we did not evaluate or exclude claims for monogenetic diabetes of the youth, which may account for a small number of the sulfonylurea or other diabetes prescriptions.

A major strength of this study is that it features a large cohort of adolescents with prediabetes to evaluate progression to T2D and on/off-label diabetes medication utilization. We excluded enrollees with PCOS, which may result in a more accurate representation of adolescent progression of prediabetes to T2D. A recent study found that 24% of patients with PCOS had a prediabetes-range HbA1c at the time of PCOS diagnosis, and that the risk of T2D is more than 18-fold greater than that of the general population of adolescents with overweight or obesity (27). However, 2,112 enrollees with PCOS were excluded, and we are unable to analyze the trajectory of these enrollees. Furthermore, to minimize external confounders, the last index date used was in 2017 to allow for 2 years of follow-up data, prior to the start of the COVID-19 pandemic.

This study was limited by a lack of laboratory, pubertal, and anthropometric data. Due to the small number of subjects prescribed diabetes medications (4.32% prescribed metformin and 0.71% prescribed second-line medication), statistical analyses were not performed. The diagnosis of prediabetes was made solely based on claims data, and it is possible the enrollees screened for prediabetes had more risk factors for T2D, biasing the population towards those more likely to be monitored and treated. The lack of laboratory data may have underestimated the true prevalence of prediabetes in this study cohort. Adult data has indicated that prediabetes is under-coded in claims data. One study of 3,888 adults with laboratory evidence of prediabetes showed that only 10.4% had a coded diagnosis of prediabetes within the following 12 months (28). Given the known high attrition rate for pediatric patients with T2D, this study classified subjects with a single HbA1C ≥ 6.5% with a diagnosis of T2D (29). A HbA1C ≥ 6.5% is diagnostic of T2D, though confirmatory testing is recommended by the United States Preventive Services Task Force (30). Although it is possible subjects were incorrectly classified as having T2D, the lack of HbA1C data overall likely underestimated the progression to T2D in this study.

The study was also confined to those with commercial claims data, without available data on race, ethnicity, and socioeconomic status. We suspect a selection bias for the subjects included in the study, as families with insurance turnover, and presumed job instability, were excluded. Our conclusions may not be accurate in patients with intermittent insurance coverage or in those with a non-commercial health insurance. We were also unable to follow the subjects for more than 2 years after prediabetes diagnosis, which may underestimate long-term risk of T2D progression. Overall, more longitudinal pediatric studies are needed on the natural history of prediabetes and on the rate of progression from prediabetes to T2D in a pediatric population.

## Conclusion

To our knowledge, this real-world experience analysis is the largest study to date on adolescents with prediabetes, showing that 2.5% of adolescent subjects with commercial claims data and prediabetes progressed to T2D over the 2-year time frame. Further longitudinal trials are needed to better evaluate T2D progression in adolescent patients with prediabetes, and to better identify and treat patients with prediabetes most at risk for development of T2D.

## Data availability statement

The raw data supporting the conclusions of this article will be made available by the authors, without undue reservation.

## Ethics statement

Ethical review and approval was not required for the study on human participants in accordance with the local legislation and institutional requirements. Written informed consent from the participants’ legal guardian/next of kin was not required to participate in this study in accordance with the national legislation and the institutional requirements.

## Author contributions

AW, MZ, SR, BT, RG, and JM contributed to the initial study design. MZ and SR performed data collection and analysis. AW drafted the manuscript. BT, RG, and JM performed critical revisions of the manuscript. All authors contributed to the article and approved the submitted version.

## Author contributions

AW, MZ, SR, BT, RG, and JM contributed to the initial study design. MZ and SR performed data collection and analysis. AW drafted the manuscript. BT, RG, and JM performed critical revisions of the manuscript. All authors contributed to the article and approved the submitted version.
